# Serotonin as a biomarker of toxin-induced Parkinsonism

**DOI:** 10.1186/s10020-023-00773-9

**Published:** 2024-03-01

**Authors:** Anna Marie Buchanan, Sergio Mena, Iman Choukari, Aditya Vasa, Jesseca N. Crawford, Jim Fadel, Nick Maxwell, Lawrence Reagan, Allie Cruikshank, Janet Best, H. Fred Nijhout, Michael Reed, Parastoo Hashemi

**Affiliations:** 1 Department of Chemistry and Biochemistry, University of South Carolina, Columbia, SC 29208 USA; 2 Department of Pharmacology, Physiology, & Neuroscience, University of South Carolina SOM, Columbia, SC 29209 USA; 3 Columbia VA Health Care System, Columbia, SC 29208 USA; 4 Department of Mathematics, Duke University, Durham, NC USA; 5 Department of Mathematics, The Ohio State University, Columbus, OH USA; 6 Department of Biology, Duke University, Durham, NC USA; 7 Department of Bioengineering, Imperial College London, London, SW7 2AZ UK

**Keywords:** FSCV, CFM, Depression

## Abstract

**Background:**

Loss of dopaminergic neurons underlies the motor symptoms of Parkinson’s disease (PD). However stereotypical PD symptoms only manifest after approximately 80% of dopamine neurons have died making dopamine-related motor phenotypes unreliable markers of the earlier stages of the disease. There are other non-motor symptoms, such as depression, that may present decades before motor symptoms.

**Methods:**

Because serotonin is implicated in depression, here we use niche, fast electrochemistry paired with mathematical modelling and machine learning to, for the first time, robustly evaluate serotonin neurochemistry in vivo in real time in a toxicological model of Parkinsonism, 1-methyl-4-phenyl-1,2,3,6-tetrahydropyridine (MPTP).

**Results:**

Mice treated with acute MPTP had lower concentrations of in vivo, evoked and ambient serotonin in the hippocampus, consistent with the clinical comorbidity of depression with PD. These mice did not chemically respond to SSRI, as strongly as control animals did, following the clinical literature showing that antidepressant success during PD is highly variable. Following L-DOPA administration, using a novel machine learning analysis tool, we observed a dynamic shift from evoked serotonin release in the hippocampus to dopamine release. We hypothesize that this finding shows, in real time, that serotonergic neurons uptake L-DOPA and produce dopamine at the expense of serotonin, supporting the significant clinical correlation between L-DOPA and depression. Finally, we found that this post L-DOPA dopamine release was less regulated, staying in the synapse for longer. This finding is perhaps due to lack of autoreceptor control and may provide a ground from which to study L-DOPA induced dyskinesia.

**Conclusions:**

These results validate key prior hypotheses about the roles of serotonin during PD and open an avenue to study to potentially improve therapeutics for levodopa-induced dyskinesia and depression.

## Background

Parkinson’s disease (PD) is a progressive neurodegenerative disorder characterized by a loss of midbrain dopaminergic neurons, eventually leading to debilitating motor deficits and death. Strikingly, dopamine neurochemistry is remarkably resilient to loss of dopamine neurons, and typical PD symptoms only manifest after approximately 80% of dopamine neurons have died. This phenomenon means that dopamine-related phenotypes are not reliable markers of the earlier stages of the disease. Early diagnosis is critical because treatments may be more effective in slowing down the progression of PD. There are a host of non-motor symptoms of PD, including depression, that precede the motor symptoms significantly (sometimes by decades). (Ishihara and Brayne 2006; Reijnders et al. 2008) This implies that there may be more salient chemical changes in response to PD onset that may mark certain phenotypes of the disease.

A modulator of interest in depression studies is serotonin (Coppen and Doogan 1988; Drevets et al. 1999; Carr and Lucki 2010). Serotonin neurochemistry is not well defined in PD models. Although not as consistent as dopamine, dysfunctions of the serotonin system have been observed in PD (Jellinger 1991; Kerenyi et al. 2003; Kish et al. 2008), with involvement at the early stages of the disease being postulated (Politis et al. 2014). There are conflicting reports of whether selective serotonin reuptake inhibitors (SSRIs) are safe (Ceravolo et al. 2000; Dell’Agnello et al. 2001) and effective (Wermuth et al. 1998; Devos et al. 2008; Menza et al. 2009; Richard et al. 2012) to treat depression in PD patients. Additionally, there is speculation that levodopa (L-DOPA) therapy exerts detrimental effects via the serotonin system as serotonin neurons may uptake this precursor to produce dopamine at the expense of serotonin (Brooks 2007; Reed et al. 2012; Carta and Tronci 2014; Politis et al. 2014; Iderberg et al. 2015; Sellnow et al. 2019; Conti Mazza et al. 2023).

Thus, we found it of great interest to explore serotonin and the effects of SSRIs and L-DOPA in a simple PD model. We combined fast-scan cyclic voltammetry (FSCV) and fast-scan controlled-adsorption voltammetry (FSCAV), niche in vivo measurement tools for serotonin, with mathematical modeling, to provide insights into these notions. We used these methods to measure and model evoked and ambient serotonin from microelectrodes in real-time in vivo in the CA2 region of the hippocampus in a toxicological model of Parkinsonism in mice. In animals treated with an acute 1-methyl-4-phenyl-1,2,3,6-tetrahydropyridine HCl (MPTP) paradigm, we found significant impairments in evoked and ambient serotonin. This finding may underlie the clinical comorbidity of depression with PD. In these same animals, an acute SSRI dose was less able to increase serotonin levels than in control mice, consistent with the notion that SSRI use during PD is highly clinically variable. We investigated L-DOPA administration by developing a novel neural network analysis that showed, in real time, rapidly after drug administration the serotonin signal was replaced by dopamine. L-DOPA is significantly associated with increased depression symptoms during PD (Hanganu et al. 2014). Our biochemical pathway models revealed the mechanism for this phenomenon. Importantly, we often found that this dopamine release was less controlled than serotonin release, potentially due to lack of autoreceptor control, which provides a serotonergic basis for L-DOPA induced dyskinesia (LID), with the limitation that we looked only at one brain region.

Thus, we provide proof of principle that the serotonin system may underlie several important facets of the MPTP model. Our work highlights the importance of serotonin and thus serotonin-related pathological phenotypes in potentially marking PD.

## Methods

### Chemicals and reagents

MPTP (16 or 18 mg kg^−1^, Sigma-Aldrich, St. Louis, MO, USA), escitalopram (Escit) oxalate (10 mg kg^−1^, Sigma-Aldrich, St. Louis, MO, USA), levodopa (50 mg kg^−1^ Sigma-Aldrich, St. Louis, MO, USA), benserazide (50 mg kg^−1^, Sigma-Aldrich, St. Louis, MO, USA) were each dissolved individually in 0.9% sterile saline (Hospira, Lake Forest, IL, USA) at a volume of 5 mL kg^−1^ animal weight and administered via intraperitoneal (*i.p.*) injection. Such a high dose of L-DOPA was administered because we only administered one acute bolus dose and in previous studies clinically relevant L-DOPA doses did not affect an FSCV dopamine signal (Qi et al. 2016). Urethane (Sigma Aldrich, St. Louis, MO, USA) was dissolved in sterile saline (0.9%, Hospira) as a 25% w/v solution and administered at 7 µL kg^−1^ via *i.p.* injection. Four-point calibration solutions were prepared by dissolving serotonin (Sigma-Aldrich) into Tris buffer to produce solutions with concentrations of 10, 25, 50, and 100 nM. Tris Buffer consisted of 15 mM Tris Buffer consisted of 15 mM H_2_NC(CH_2_OH)_2_ HCl, 140 mM NaCl, 3.25 mM KCl, 1.2 mM CaCl2, 1.25 mM NaH_2_PO_4_ ·H_2_O, 1.2 mM MgCl_2_, and 2.0 mM Na_2_SO_4_ (Sigma-Aldrich).

### Electrode fabrication

Carbon fiber microelectrodes were fabricated by aspirating a carbon fiber (Goodfellow Corporation, Coraopolis, PA, United States) into a glass capillary (0.4 mm internal diameter, 0.6 mm outer diameter, AM Systems, Carlsborg, WA, United States). Electrodes were pulled to a fine tip using a vertical pipette puller (Narishige Group, Tokyo, Japan), creating a carbon-glass seal. The exposed carbon fiber was then cut to 150 µm and Nafion (L-Q-1105, Ion Power, New Castle, DE, USA) was electroplated onto the electrode surface by applying a constant potential (~ 1 V) for 30 s.

### Behavioral testing procedure

Control and MPTP-treated animals underwent previously established behavioral testing (Krishnan et al. 2007). Behavior of animals was analyzed using Noldus EthoVision (Leesburg, VA, USA). The elevated zero maze test was performed as reported before (Tucker and McCabe 2017). Mice were placed into the closed section of the maze (Maze Engineers, Boston, MA, USA) and were allowed to explore for 5 min. Time spent in the closed section was then measured and reported as a percentage of the total time. The tail suspension test was completed as previously described (Sanna et al. 2017). Mice were attached using tape to a rod and a flexible tube was placed on the tail to limit climbing behavior within the apparatus (Maze Engineers, Boston, MA, USA) for the duration of the test (6 min). The total immobility time was then measured and reported. The open field test was also performed as described earlier (Gould et al. 2009). Mice were placed in an 35 × 35 × 35 cm activity chamber (Maze Engineers, Boston, MA, USA) and their total distance travelled was measured for 20 min. On the two days prior to the test, the animals were habituated to the field for 10 min.

### Behavioral exclusion criteria

Tail Suspension Test Mice were excluded from analysis in the tail suspension test if they fell from the apparatus or climbed their tails to the extent that they were able to grasp the metal bar they were suspended from. Elevated Zero Maze Animals that fell from the apparatus were excluded from analysis. Open Field Test Any animals that escaped the open field area were excluded.

### Animals and surgical procedure

All animal procedures and protocols were performed in accordance with regulations of the Institutional Animal Care and Use Committee (IACUC) at the University of South Carolina, which operates with accreditation from the Association for Assessment and Accreditation of Laboratory Animal Care (AAALAC). Male and female C57BL/6 J mice at 10–14 weeks of age were group housed on a 12 h light dark cycle with ad libitum access to food and water. At 10–11 weeks of age, mice underwent an acute MPTP injection paradigm (4 doses every 2 h, *i.p.*) (Jackson-Lewis and Przedborski 2007). Mice were injected with MPTP hydrochloride, while control mice were administered an equal volume of saline. Male mice received a dose of 18 mg kg^−1^ MPTP and female mice received a dose of 16 mg kg^−1^ MPTP, a dose more easily tolerated by the smaller female mice. The lesions were allowed to stabilize 7 days before surgeries were performed.

Following the administration of urethane anesthesia via* i.p.* injection, stereotaxic surgery was performed with all coordinates taken in reference to bregma. For serotonin measurements, the CFM was lowered into the CA2 region of the hippocampus (AP: − 2.91, ML: + 3.35, DV: − 2.5 to 3.2) and compared to a pseudo-Ag/AgCl reference electrode placed in the contralateral hemisphere. A stimulating electrode (insulated stainless steel, diameter: 0.2 mm, untwisted, Plastics One, Roanoke, VA, United States) was placed into the medial forebrain bundle (AP: − 1.58, ML: + 1.00, DV: − 4.8). For dopamine measurements, the CFM was lowered until it was fully immersed in the dorsal striatum (AP: + 1.1, ML: + 1.7, DV: − 3.0), and dopamine release was also electrically evoked placing the electrode in the medial forebrain bundle (AP: − 1.8, ML: + 1.1, DV:− 4.8). A thermal heating pad (Braintree Scientific, Braintree, MA, USA) was used to maintain mouse body temperature for the duration of the experiment.

### Data collection and analysis

FSCV was performed on anesthetized mice to measure phasic serotonin release and reuptake as previously described (Hashemi 2009; Saylor 2019). All measurements were collected using a Dagan potentiostat (Dagan Corporation, Minneapolis, NM), custom built hardware interfaced with PCIe 6431 and PCI 6221 DAC/ADC cards (National Instruments, Austin, TX), and a Pine Research headstage (Pine Research Instruments, Durham, NC). WCCV 3.06 software (Knowmad Technologies LLC, Tucson, AZ) was used to apply the serotonin waveform (0.2 V to 1.0 V to − 0.1 V to 0.2 V) at a scan rate of 1000 V s^−1^. The waveform was cycled at a frequency of 60 Hz for 10 min, then at 10 Hz for 10 min prior to data acquisition. The same waveform was applied for dopamine measurements in the striatum. A biphasic electrical stimulation (60 Hz, 360 µA, 2 ms in width) was applied for 2 s through a linear constant current stimulus isolator (NL800A Neurolog, Medical Systems Corp, Great Neck, NY) to evoke serotonin release.

Data was collected and filtered using WCCV software (zero phase, Butterworth, 3 kHz low pass filter). Four control measurements, taken every 10 min, were collected and averaged. Then either Escit (10 mg kg^−1^) or a combination of L-DOPA (50 mg kg^−1^) and benserazide (50 mg kg^−1^) were administered. Measurements were taken at the 0 min, 5 min, and 10 min time points and then once every additional 10 min for 2 h following drug administration. The obtained currents were converted to concentration using a previously reported calibration factor (49.5 nM nA^−1^) (Hashemi et al. 2009).

FSCAV was utilized to measure the ambient concentrations of serotonin (Abdalla et al. 2017) in vivo using the same hardware as FSCV. Control evoked serotonin measurements were collected prior to switching to basal collection. For FSCAV collection, the serotonin waveform was applied at a frequency of 100 Hz for 2 s to minimize adsorption to the electrode surface. This was followed by a period of controlled adsorption where the electrode is held at a constant potential (0.2 V) for a period of 10 s. Finally, the waveform was reapplied at 100 Hz and the first CV characteristic of serotonin was selected. Control measurements were collected once a minute for 30 min. Following control measurements, saline was administered *i.p.* (5.0 mg kg^−1^) and files were taken for an additional 30 min. Animals were then administered Escit (10 mg kg^−1^*, i.p.*) and 60 files were collected. The peak observed between 0.4 V and 0.8 V was integrated to obtain a charge (pC) and following removal from the brain, a post calibration of each electrode was used to determine concentration (solutions of 10, 25, 50, and 100 nM serotonin in Tris buffer).

### Measurement methods

**Fig. 1:**
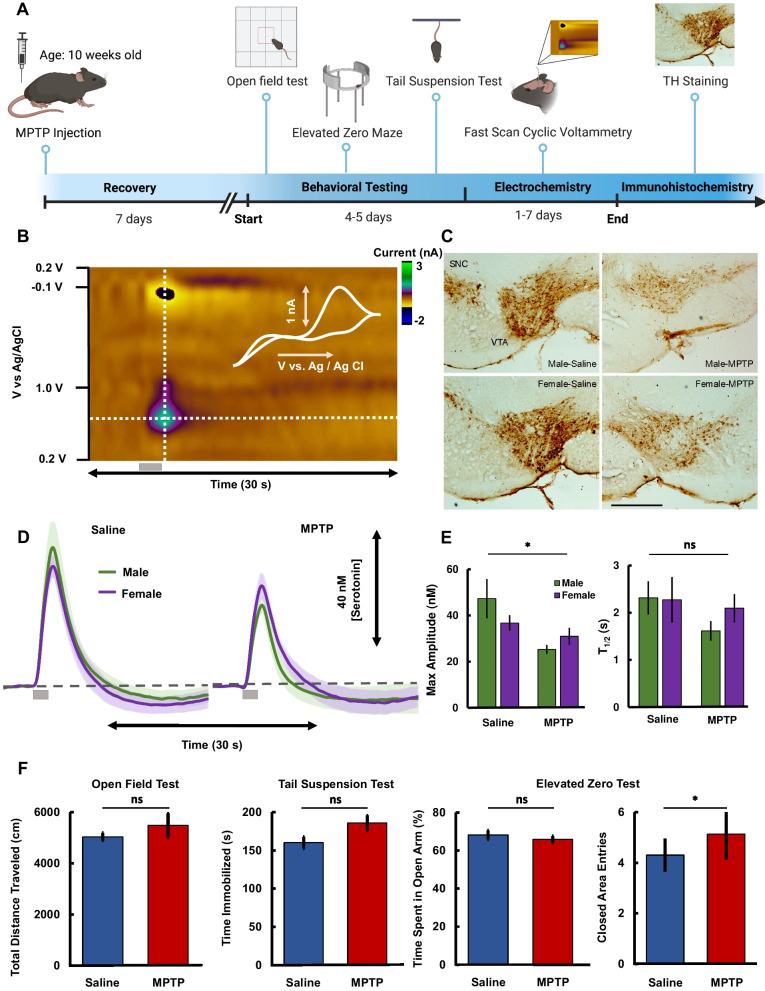
**A** Schematic of experimental paradigm. **B** Representative color plot in a mouse hippocampus depicting serotonin oxidation in an animal after MPTP. Vertical line shows the CV overlaid in the right-hand corner. Horizontal line shows the concentration *vs.* time (IT) curves presented in D. **C** Representative images of tyrosine hydroxylase immunostaining in male (top images) and female (bottom images) mice administered either with saline vehicle (left images) or MPTP (right images). Scale bar is 250 µm in length. **D** Concentration vs. time curve (average ± SEM) comparing mice administered MPTP (n = 22, 13 male and 9 female) *vs.* mice administered saline (n = 15, 5 male and 10 female). Male is shown in green while females are shown in purple. **E** Comparison of max amplitudes and serotonin reuptake decay constant (average ± SEM) of FSCV curves presented in **D**. **F** Comparison of test scores between control mice (blue) and MPTP-treated mice (red) (tail suspension test: n = 14 control, n = 18 MPTP-treated; open field test: n = 10 control, n = 12 MPTP-treated; elevated zero maze test, n = 17 control, n = 22 MPTP-treated)

Maximum amplitude of evoked release was measured for each individual repetition using custom-designed peak finding algorithms. Area under the curve was measured using the Simpson’s rule. Half-life of the decay trace was measured by fitting an exponential decay curve after the maximum amplitude of the release (Mena et al. 2021). A previously described Michaelis–Menten model with two reuptake processes (Eq. 8) (Wood et al. 2014) was fitted to the average FSCV trace of each group using a custom-designed gradient descent algorithm. The first uptake kinetic parameters, associated with serotonin transporters, were allowed to fit the experimental signals, while the second uptake mechanism was kept constant. We fit the model to the average trace. This is because the ambient noise inherent in individual traces renders the models inaccurate since the model is very sensitive to overfitting. Averaging the traces removes noise and drift and thus the models are much more accurate. We additionally highlight another possible limitation: uptake 2 kinetic parameters are held constant under the rationale that the number of uptake 2 transporters does not change. Our experimental data in Fig. 1D and E show no differences in t_1/2_, supporting our assumption.

### Immunohistochemistry

Following euthanasia, representative free-floating coronal sections through the rostrocaudal extent of the ventral midbrain were incubated in monoclonal mouse α-tyrosine hydroxylase (TH; 1:3000; Immunostar, Hudson, WI; Lot# 907001; Cat.#22941) followed by biotinylated donkey anti-mouse IgG (1:1000; Jackson ImmunoResearch, West Grove, PA) and horseradish peroxidase-conjugated streptavidin (1:1600; Jackson ImmunoResearch). TH immunolabeling was visualized by the addition of 0.3% hydrogen peroxide to sections incubated in 3% diaminobenzidine solution, resulting in brown labeling confined to the cytoplasm of TH-positive (putatively dopaminergic) neurons in the VTA and substantia nigra. Density of TH labeling was used as a qualitative indication of MPTP lesion efficacy. A 250 × 250 µm area of the staining was extracted from each image, converted to grayscale, and opacity of the staining was then measured as inverse of the brightness of the selected section.

### Statistical analyses

**Fig. 2:**
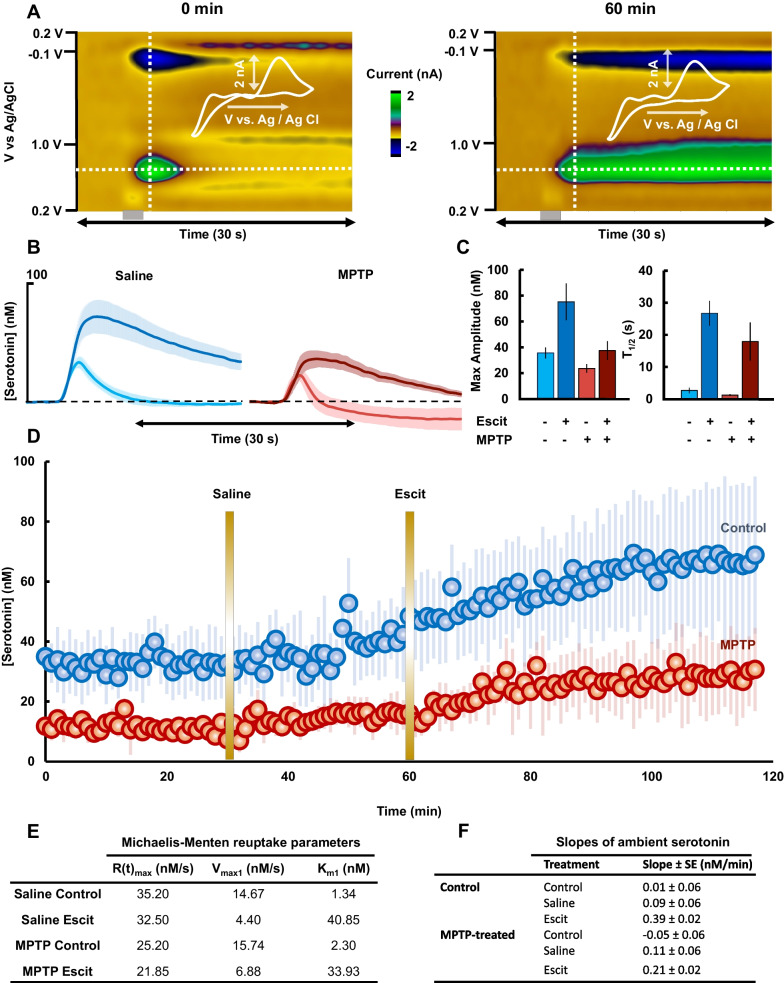
**A** Representative color plot depicting serotonin oxidation before (left) and after Escit administration (right) in a MPTP-administered mouse (hippocampus). Vertical line shows the CV overlaid in the right-hand corner. Horizontal line shows the concentration vs time curves presented in B. **B** Concentration vs. time curve comparing mice administered saline before (light blue) and 60 min after (dark blue) Escit (10 mg kg^−1^) administration (n = 5) and MPTP-administered mice before (light red) and after (dark red) Escit (10 mg kg^−1^) administration (n = 5). **C** Comparison of *Amp*_*max*_ and *t*_1/2_ of FSCV curves presented in B. Saline animals evoke significantly higher serotonin than MPTP-administered mice before (*post-hoc t*-test, Amp_max_ = 35.21 ± 2.17 nM *vs.* 23.56 ± 1.70 nM, p = 0.0336) and after Escit (*post-hoc* test, Amp_max_ = 75.22 ± 14.24 nM *vs.* 37.46 ± 7.32 nM, p = 0.0095. No statistical significance was found in the reuptake rate of evoked serotonin between saline and MPTP-administered animals before (*post-hoc t*-test, t_1/2_ = 2.47 ± 0.82 s *vs.* 1.24 ± 0.31 s, p = 1.000) or after Escit administration (*post-hoc* test, t_1/2_ = 26.70 ± 3.89 s *vs.* 17.92 ± 5.96 s, p = 0.7691). **D** Average (with SEM as error bars) ambient concentrations of serotonin collected using FSCAV before and after Escit administration for MPTP (red dots, n = 5) and saline (blue dots, n = 5) animals. **E** Michaelis–Menten kinetic parameters fit to the time traces in **B**. **F** ANCOVA slopes and standard error of the slopes of the time series in **D**

Statistical significance is defined as p < 0.05. All statistical tests are performed using Matlab 2020b. Sample distributions are described as mean ± SEM if not stated otherwise. A total of 22 mice were pre-treated with MPTP, consisting of 13 males and 9 females. Additionally, 15 mice were treated with saline, with 5 males and 10 females in this group (Fig. 1). After the application of exclusion criteria (vide supra), 14 control mice and 18 MPTP-treated animals were included in the tail suspension test. In the open field test, 10 control mice and 12 MPTP-treated animals were included. Lastly, the elevated zero maze test involved 17 control mice and 22 MPTP-treated animals. Five MPTP-treated mice and 5 control mice were *i.p.* injected with saline and Escit (Fig. 2). Five MPTP-treated mice and 5 control mice were *i.p.* injected with (L-DOPA), while another cohort of 5 control mice were injected with saline (Fig. 4). Differences in behavioral scores were tested using two-way analysis of variance (ANOVA) and *Tukey–Kramer post-hoc* multiple comparisons with factors of sex of mice and MPTP-treatment. Differences in maximum amplitude and reuptake rate between groups were tested for significance using two-way ANOVA, mixed ANOVA and *Tukey–Kramer post-hoc* multiple comparisons. For the two-way ANOVA, the effects accounted were sex of mice or time since MPTP administration (7–14 or 15–21 days), and the treatment applied (MPTP-treated or saline-treated). For the mixed ANOVA, the two effects considered were the type of mice (MPTP-treated or saline-treated) and time after Escit administration. Differences in basal concentrations of serotonin and the predicted relative ratios of serotonin to dopamine after L-DOPA treatment were also tested for significance using mixed ANOVA, ANCOVA and *Tukey–Kramer post-hoc* multiple comparisons. For the absolute predictions of serotonin, the two effects were the type of mice (MPTP-treated or saline-treated) and the different treatments during basal experiment (control, saline and Escit separated in time). For the ratio of serotonin to dopamine, the two effects were the type of mice (MPTP-treated or saline-treated) and the time after L-DOPA injection. Other statistical results are available upon request.

### Data augmentation

Data augmentation of in vivo acquisitions was performed to generate synthetic data as a combination of two random control acquisitions (color plots) known to be representative of a serotonin evoked release (drawn from the CA2 region of the hippocampus) and dopamine evoked release (drawn from the striatum). The operation to obtain the synthetic color plots is expressed in Eq. 1

1 $$\begin{array}{c}{X}_{k}={r}_{k}\cdot A+\left(1-{r}_{k}\right)\cdot B\end{array}$$

where ${X}_{k}$ is the new synthetic color plot, ${r}_{k}$ is the randomized ratio from 0 to 1, and A and B are the serotonin and dopamine control color plots from their respective regions of the brain, respectively. All the color plots used to generate synthetic data are standardized to have a mean of zero and standard deviation of unity to prevent artifacts due to differences in current amplitude of the color plots. Five thousand color plots were generated per animal with randomized ratio, serotonin and dopamine acquisitions to be used as training and validation set for the neural network models.

### Convolutional neural networks

Convolutional neural networks (CNN) were designed, trained and deployed using Tensorflow and Keras in Python 3.9.2 (Abadi et al. 2016) The CNN were designed to function as regression models; the final layer of the model consists of a unity node which predicts a relative ratio of serotonin to dopamine from the two-dimensional color plot. The predicted ratio of serotonin to dopamine is relative to dopamine measurements in the striatum of rodents (assumed to have a ratio of zero) and serotonin measurements in the CA2 region of the hippocampus of rodents (assumed to have a ratio of one). The CNN trained in this work consisted of 11 layers: two convolution layers, one max pooling layer, four leaky rectified linear unit (ReLU) layers, a flatten layer after the set of convolution layers and three deeply connected (dense) layers. All layers were integrated in a sequential model. The first hidden layer consisted of a convolution layer (275 features), which computes the Hadamard product of a kernel of 3-by-3 with each of the input values of the color plot. This is followed by a scalar exponential linear unit (SELU) activation, which follows the mathematical function shown in Eq. 2 (Abadi et al. 2016; Ding et al. 2018).

2 $$\begin{array}{c}f (x) = \left\{\begin{array}{c} \qquad\qquad s\cdot x, \quad x>0\\ s\cdot{(\alpha\cdot e}^{x} - 1), \quad x\le 0\end{array}\right.\end{array}$$

where s and α are predefined constants (s = 1.051 and α = 1.673). Data dimensionality and overfitting were reduced with the use of a succeeding max pooling layer (*i.e.*, down sampling the input along its spatial dimensions by taking the maximum value over an input window of size 3-by-3). A third layer with leaky ReLU was then applied following the mathematical expression in Eq. 3 (Abadi et al. 2016)

3 $$\begin{array}{c}f (x) = \left\{\begin{array}{c} \qquad x, \quad x>0\\ 0.1\cdot x, \quad x\le 0\end{array}\right.\end{array}$$

**Fig. 3:**
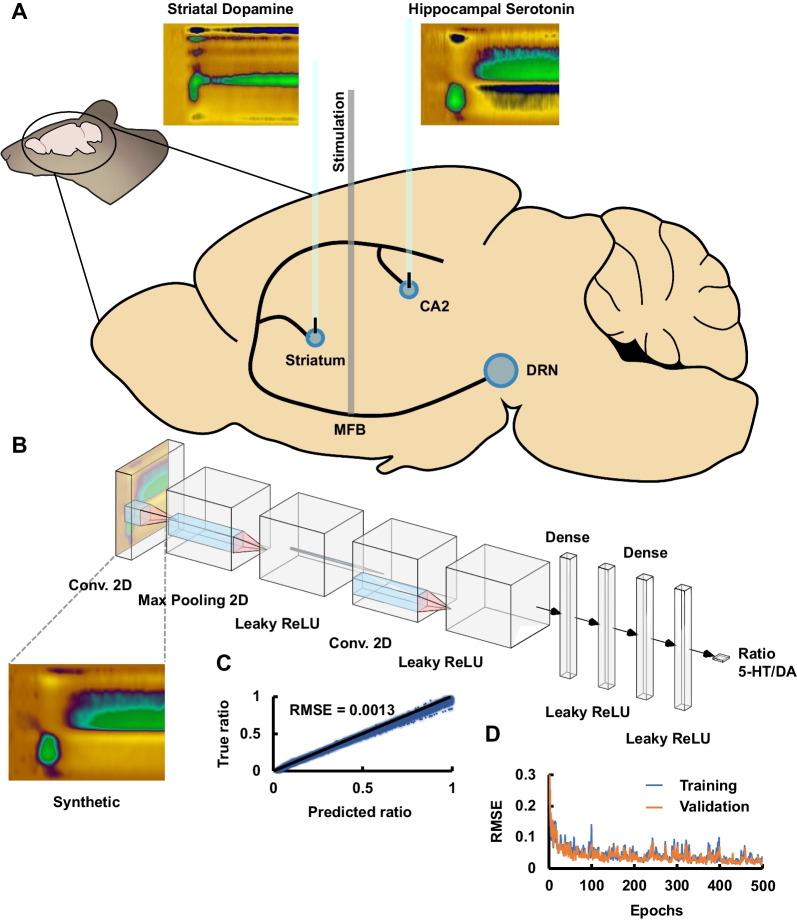
**A** Schematic of the mouse brain and locations of serotonin-rich and dopamine-rich signals drawn to generate the synthetic color plots. **B** CNN architecture schematic. **C** True *vs.* predicted values of the test dataset and RMSE of predicted values. **D** Training and validation loss (RMSE) of CNN training

The fourth and fifth layers consisted of a consecutive convolutional layer (100 features and ReLU activation) and leaky ReLU activation function layer. The structure of the CNN was then flattened and a set of dense and leaky ReLU were applied to the one-dimensional input stream. The CNN finishes with a unity output node which predicts the relative ratio of serotonin to dopamine. Figure 3B shows the schematic structure of the CNN. The model was trained with 5000 synthetic control color plots and ratios. Each synthetic color plot is generated from a randomized combination of a hippocampal serotonin (n = 7 mice, 4 repetitions per animal, 28 color plots) and striatal dopamine color plot (n = 3 mice, 7 repetitions per animal, 21 color plots), and Gaussian noise is used at the input of the CNN so that each signal is unique. Training and test splits were set to a 4:1 ratio. Training batches were set to 30 shuffled color plots and train and validation splits were set as 4:1 ratio with 4 steps per iteration and 5 validation steps (k-fold cross-validation). The model was trained using the Adam optimizer (Kingma and Ba 2014) with a learning rate of 0.0001 and mean absolute error as the loss function. The root mean square error (RMSE) between predicted and true ratios was used to assess the predictive power of the neural network. Additionally, 500 iterations were used as higher values did not improve the predictive error of the regression. Similarly, fewer iterations were found to reduce the performance. After training, unseen acquisitions after L-DOPA administration were used to predict the change of relative ratio of serotonin and dopamine.

### Mathematical modelling of serotonin and dopamine interaction

We used the mathematical model created in (Reed et al. 2012) to study the interaction of serotonin and dopamine in the hippocampus. Detailed description of the model can be found in the original publication, and only the additions are described here. Originally, the model was used to reflect the striatum, but since both dorsal raphe nucleus (DRN) and substantia nigra pars compacta (SNc) project to the hippocampus (Guiard et al. 2008; Monti 2010), the model can equally be used for the hippocampus. The leakage rate out of the serotonin vesicles was changed from 0.4 to 40 h^−1^ to bring down the concentration of serotonin in the model to 35 nM, as observed in the experimental results. Additionally, we added stimulation of the DRN by the SNc (Guiard et al. 2008), which follows the expression in Eq. 4 below.

4 $$\begin{array}{c}stimulation=f\cdot \left(1.5-\left(\frac{{c}^{2}}{{0.000968}^{2}+{c}^{2}}\right)\right)\end{array}$$

where $f$ is the fraction of SNc cells still alive and 0.000968 and *c* represent the equilibrium and actual concentration of serotonin in the DRN (in µM), respectively. The model assumes that all the stimulation of the DRN neurons that project to the hippocampus comes from the SNc.

Escit effects to serotonin hippocampal concentration (Fig. 2) were modelled adding 3 variables to the model: $E(t)$, the concentration of Escit in the extracellular space, $F(t)$, the concentration of free serotonin transporters (SERTs), $B(t)$, the concentration of bound SERTs. The differential equations satisfied by the variables are given in Eqs. 5, 6 and 7 below.

5 $$\begin{array}{c}\frac{dE}{dt}=I\left(t\right)-0.371\cdot E\left(t\right)\end{array}$$

6 $$\begin{array}{c}\frac{dF}{dt}={k}_{2}\cdot B\left(t\right)-{k}_{1}\cdot E\left(t\right)\cdot F\left(t\right)\end{array}$$

7 $$\begin{array}{c}\frac{dB}{dt}={k}_{1}\cdot E\left(t\right)\cdot F\left(t\right)-{k}_{2}\cdot B\left(t\right)\end{array}$$

We choose $I(t)$, which is the input of Escit from the blood to the extracellular space, and the coefficient 0.371, so that $E(t)$ has the same shape as the curve measured and computed in (Bundgaard et al. 2007), who studied transport of Escit across the blood–brain barrier. We choose the amplitude of *I(t)* and the rate constants ${k}_{1}$ of 7 and ${k}_{2}$ of 20 so that, at maximum, 50% of the SERTs are occupied by Escit. Additionally, MPTP effects on extracellular serotonin were modelled assuming that only 35% of the SNc dopamine neurons remained alive.

## Results

### Evoked serotonin in an acute toxicological model of PD

A well accepted, acute toxicological model of Parkinsonism was employed in mice. In brief, MPTP was administered intraperitoneally (*i.p*). in male and female mice four times, once every two hours. After a 7–21 day wait period (Fig. 1A shows the entire experimental paradigm) for the lesions to stabilize, animals underwent behavioral testing. One cohort of animals underwent testing for motor deficits using the open field test (Fig. 1F), no significant differences were found between control and MPTP-treated animals (two-way ANOVA, distance = 50.34 ± 1.52 m *vs.* 54.80 ± 4.67 m, F = 0.64, p = 0.4344). Another cohort of animals underwent behavioral testing for depressive and anxiolytic-like phenotypes, and no significant differences were found between control and MPTP-administered mice using the tail suspension test (two-way ANOVA, immobility = 160.14 ± 7.64 s *vs.* 185.78 ± 9.96 s, F = 2.83, p = 0.1034), elevated zero maze test (two-way ANOVA, percentage time in closed arms = 68.16 ± 2.39 *vs.* 65.79 ± 1.76, F = 1.22, p = 0.2767; number of entries to closed arms = 3.71 ± 0.65 vs. 5.00 ± 0.74, p = 0.0143). Next, in these same mice, evoked serotonin was measured with FSCV in the CA2 region of the hippocampus. Figure 1B shows a representative FSCV color plot of the serotonin signal in a MPTP-treated mouse. Briefly, this two-dimensional plot (voltage *vs.* time), with current in false color, represents serotonin oxidation and reduction (vertical line). The inset cyclic voltammogram (CV) (upper right-hand corner) can be extracted from this vertical section and identifies serotonin via the unique positions of the oxidation and reduction peaks. The current *vs.* time curves are from a horizontal section (horizontal dotted line) and depicts how the analyte concentration changes over time (Fig. 1D).

In 22 mice that underwent the MPTP paradigm, in the hippocampus, maximum evoked serotonin (Amp_max_) was significantly lower than in saline-treated mice (Amp_max_ = 27.70 ± 2.04 nM *vs.* 40.20 ± 5.34 nM, two-way ANOVA, F = 7.40, p = 0.0103), no difference was seen in the rate of serotonin reuptake between MPTP (t_1/2_ = 1.79 ± 0.19 s) and saline (t_/12_ = 2.27 ± 0.33 s) animals (two-way ANOVA, F = 1.39, p = 0.2464). The average traces were modeled with a previously described 2-reuptake Michaelis Menten model to evaluate serotonin kinetics (uptake 1 represents SERTs and uptake 2 represents the other monoamine transporters). We previously used Eq. 8 (Wood et al. 2014) to model these two reuptake mechanisms:

8 $$\begin{array}{c}\frac{dC\left(t\right)}{dt}=R\left(t\right)\left(1-A\left(t\right)\right)-\alpha \frac{{V}_{max1}\cdot C\left(t\right)}{{K}_{m1}+C\left(t\right)}-\beta \frac{{V}_{max2}\cdot C\left(t\right)}{{K}_{m2}+C\left(t\right)}\end{array}$$

C(t) is the concentration of the neurotransmitter in the extracellular space, R(t) is the evoked release rate of the neurotransmitter, A(t) is the fraction of occupied autoreceptors of the neurotransmitter, which works as negative feedback control, α and β are the weights of the two reuptake mechanisms, and V_max_ and K_m_ are Michaelis Menten reuptake parameters. The reuptake parameters did not differ between control and MPTP–administered mice (MPTP: V_max1_ = 12.15 nM s^−1^, K_m1_ = 2.61 nM, V_max2_ = 780 nM s^−1^, K_m2_ = 170 nM; saline: V_max1_ = 11.13 nM s^−1^, K_m1_ = 2.40 nM, V_max2_ = 780 nM s^−1^, K_m2_ = 170 nM). A notable change was observed in maximum release rate in MPTP mice (R(t) = 27.20 nM s^−1^) compared to saline controls (R(t) = 38.40 nM s^−1^), suggesting a decreased neuronal output in MPTP animals. The MPTP-induced decrease in serotonin was not found to be significantly different between male and female mice in either maximum amplitude of release (two-way ANOVA, Amp_max_ = 23.70 ± 2.26 nM *vs.* 31.09 ± 4.03 nM, F = 0.90, p = 0.3554) or reuptake (two-way ANOVA, t_1/2_ = 1.60 ± 0.21 s *vs.* 2.07 ± 0.34 s, F = 0.37, p = 0.5518) (Fig. 1E). Additionally, the toxin-induced decrease in maximum amplitude did not significantly change between 7–13 and 14–21 days after injection (data not shown in figure) (two-way ANOVA, Amp_max_ = 23.68 ± 2.41 nM *vs.* 30.36 ± 3.72 nM, F = 0.17, p = 0.6865).

Post-experiment, brains were fixed, sliced and stained for tyrosine hydroxylase (a well-established marker MPTP efficacy) (Wulle and Schnitzer 1989). Representative images of brain slices (control and MPTP) showing the ventral tegmental area (VTA) and SNc are shown in Fig. 1C. In the MPTP brain slices, visually, there were consistently fewer dopamine neurons after MPTP, evidenced by an increase in opacity of staining after MPTP administration for female (9.95 a.u. µm^−2^ *vs.* 6.95 a.u. µm^−2^) and male (8.82 a.u. µm^−2^ *vs.* 6.37 a.u. µm^−2^) mice. Due to low n number of post-experiment animals, we could not perform statistical analysis on quantitative MPTP-induced dopaminergic cell loss.

### Escitalopram administration in MPTP-treated mice

In this experiment we administered Escit (10 mg kg^−1^) to MPTP-treated and control mice. Representative color plots for serotonin oxidation/reduction in the CA2 region of the hippocampus in MPTP animals are presented in Fig. 2A. Here is shown a serotonin signal in the CA2 region of the hippocampus in a MPTP-treated mouse along with the same signal 60 min after Escit. The concentration *vs.* time curves are extracted from the horizontal dashed lines, averaged between animals, and represented in Fig. 2B.

Both the application of MPTP and Escit have an effect on maximum amplitude of the signal (mixed ANOVA, MPTP factor: F = 7.31, p = 0.0269; Escit factor: F = 16.83, p < 0.0001) but on reuptake rate, only the application of Escit has a significant effect (mixed ANOVA, MPTP factor: F = 0.94, p = 0.3606; Escit factor: F = 20.53, p < 0.0001). In control mice, Escit administration increased the maximum release amplitude from 35.21 ± 2.17 nM to 75.22 ± 14.24 nM (*post-hoc* paired* t*-test, p = 0.0386), and the t_1/2_ of reuptake from 2.47 ± 0.82 s to 26.70 ± 3.89 s after 60 min (*post-hoc* paired* t*-test, p = 0.0056). In MPTP-treated mice, the amplitude increased from 23.56 ± 1.70 nM to 37.46 ± 7.32 nM (*post-hoc* paired* t*-test, p = 0.1175) and the t_1/2_ increased from 1.24 ± 0.31 s to 17.92 ± 5.96 s, (*post-hoc* paired* t*-test, p = 0.0287). These values are compared in Fig. 2C. Next, we evaluated Michaelis Menten kinetics for the average curves. V_max1_ (maximum rate of SERTs), K_m1_ (substrate concentration at V_max1_/2) and R(t)_max_ (maximum rate of serotonin release) were fitted to the experimental data. R(t)_max_ in control mice (35.20 nM s^−1^) decreased to 32.50 nM s^−1^ after Escit. R(t)_max_ in MPTP was 25.20 nM s^−1^ before Escit, decreasing to 21.85 nM s^−1^. V_max1_ in saline controls decreased from 14.67 to 4.40 nM s^−1^ (~ 70% decrease) 60 min after Escit, while K_m1_ increased from 1.34 to 40.85 nM. In MPTP-treated mice, after Escit V_max1_ decreased from 15.74 to 6.88 nM s^−1^ (~ 60% decrease), while K_m1_ increased from 2.30 to 33.93 nM. These values are all tabulated in Fig. 2E.

To measure ambient serotonin concentrations, we utilized FSCAV (Fig. 2D) and measured extracellular serotonin once a minute for 120 min (Atcherley et al. 2013, 2015; Burrell et al. 2015; Abdalla et al. 2017). Both the application of MPTP and Escit have an effect on basal levels of serotonin (mixed ANOVA, MPTP factor: F = 257.22, p < 0.0001; Escit factor: F = 2.89, p < 0.0001). During a control period of 30 min, ambient serotonin concentrations were 32.65 ± 1.88 nM in control mice and 11.46 ± 0.60 nM in MPTP-treated mice (*post-hoc t-test,* p < 0.0001). This shows that MPTP treatment also significantly reduces ambient serotonin levels. After 60 min, Escit was administered to both animal groups; serotonin concentrations were raised significantly 60 min after this (Control: 32.65 ± 1.88 nM to 65.34 ± 19.61 nM, *post-hoc* paired *t-test*, p = 0.0269 (+ 32.69 nM); MPTP: 11.46 ± 0.60 nM to 26.69 ± 10.14 nM, *post-hoc* paired *t-test,* p = 0.0243 (+ 15.23 nM)). We then compared the slopes of the average traces with analysis of covariance (ANCOVA). The slopes of the control and saline traces are not significantly different in both animal groups (Control: 0.01 ± 0.06 nM min^−1^ *vs.* 0.09 ± 0.06 nM min^−1^, *post-hoc t-test*, p = 0.2441; MPTP: − 0.05 ± 0.06 nM min^−1^ *vs.* 0.11 ± 0.06 nM min^−1^, *post-hoc t-test*, p = 0.3229). Slopes after Escit administration are significantly different respect to the control state in both animals (Control: 0.01 ± 0.06 nM min^−1^ *vs.* 0.39 ± 0.02 nM min^−1^, *post-hoc t-test*, p < 0.0001; MPTP: − 0.05 ± 0.06 nM min^−1^ *vs.* 0.21 ± 0.02 nM min^−1^, *post-hoc t-test*, p = 0.0002). Importantly, there is a statistical difference in the slope after Escit between control and MPTP-treated mice (0.39 ± 0.02 nM min^−1^ vs. 0.21 ± 0.02 nM min^−1^, *post-hoc t-test*, p < 0.0001). Figure 2F shows these values in a table.

### L-DOPA administration in MPTP-mice

We next wanted to ask about the effects of L-DOPA administration on the serotonin signal. We hypothesized that dopamine might be co-released after this treatment, however because the dopamine and serotonin FSCV signals overlap, to test this hypothesis we needed to take a novel data analysis approach.

**Fig. 4:**
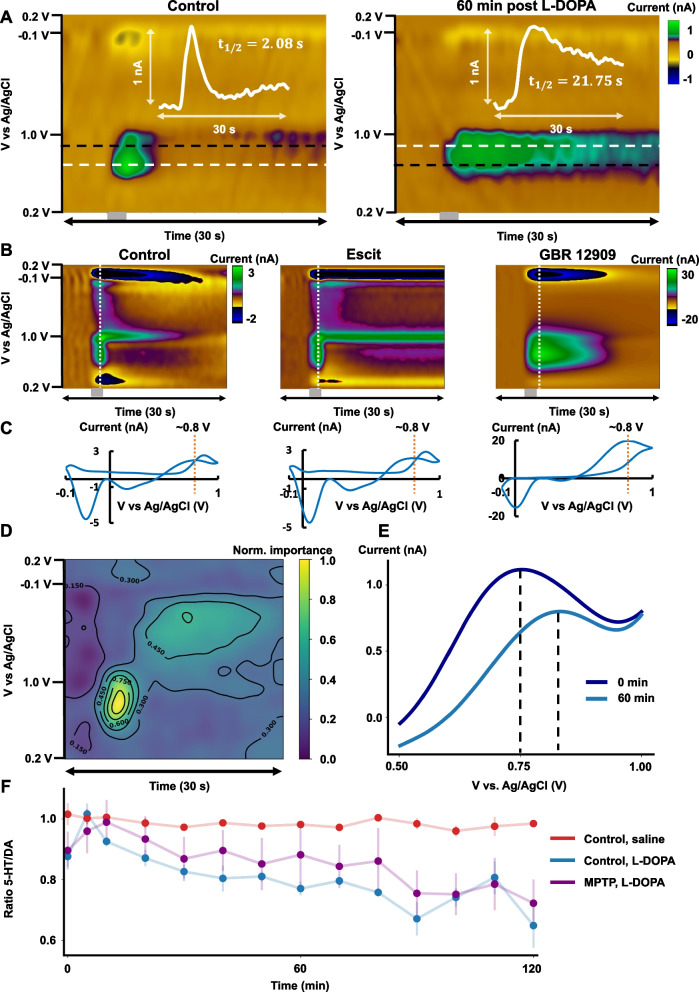
**A** Left: Representative color plot depicting serotonin oxidation before L-DOPA administration in an MPTP-administered mouse (hippocampus). Horizontal line (white) shows the shift in the center of the serotonin oxidation. Right: Representative color plot depicting serotonin oxidation 60 min after L-DOPA (50 mg kg^−1^, *i.p*) administration. **B** Dopamine detection in the striatum with the serotonin waveform after Escit and GBR 12909. **C** Representative CVs from dopamine color plots in B. **D** Normalized, averaged (n = 15 repetitions) importance of the features in the FSCV color plot. **E** Representative voltammograms from A show a shift in oxidation potential, 60 min after L-DOPA administration. **F** Average ± SEM predicted ratios over time after injection of saline over control mice (red trace, n = 3), or L-DOPA (50 mg kg^−1^) for control mice (blue trace, n = 5) and MPTP-treated mice (purple trace, n = 5)

We took original in vivo acquisitions of dopamine in the striatum (with the serotonin waveform, and verified it was dopamine using a dopamine transporter (DAT) inhibitor, GBR 12909, see Figs. 3A and 4B) and serotonin in the hippocampus and applied data augmentation to generate labeled synthetic in vivo color plots (Fig. 3B) (a randomized relative ratio of serotonin from the CA2 region to dopamine from the striatum) using the mathematical expression shown in Eq. 2.

A CNN (Fig. 3B) was then designed and trained using the synthetic signals to predict the relative ratio from the whole color plot. The structure of the neural network was optimized to improve the performance of the CNN in the prediction of the ratio from unseen test data. A low training and validation loss, as well as predictive test error [RMSE] was achieved using 500 epochs (Fig. 3C and D). After the training, the neural network was used to predict the relative ratio of serotonin to dopamine. Figure 3B shows the schematic of the final CNN model after optimization of the design. Briefly, the function of the model is to select features from the color plot (via convolution) that contribute to determine whether the acquisition resembles serotonin release (such as those found in the CA2 region of the hippocampus) or dopamine release (such as those found in the striatum). Here we used our CNN to identify changes from serotonin-rich release to a dopamine-rich release and then used the model to obtain a prediction of a relative ratio of serotonin to dopamine in the CA2 region of the hippocampus after L-DOPA (50 mg kg^−1^, *i.p*.) administration with time.

Figure 4A shows representative color plots from an MPTP-treated mouse before and after L-DOPA; the shift in oxidation potential is clear (and verified with the voltammograms in Fig. 4E), implying a shift from serotonin to dopamine release.

Additionally, the current *vs.* time traces embedded in the color plots show a change in the reuptake rate of the neurotransmitter after L-DOPA administration. This change in the reuptake was evident in 2 out of the 5 mice (t_1/2_ = 2.08 s *vs.* 21.75 s and t_1/2_ = 1.56 s *vs.* 10.72 s) that were administered MPTP and L-DOPA**,** while none of the saline-administered animals treated with L-DOPA had a significant change in their reuptake profile. Figure 4B and C shows dopamine signals in the striatum with the serotonin waveform. The control signal (left) did not respond to Escit (10 mg kg^−1^, middle) but did respond to GBR 12909 (10 mg kg^−1^, right) confirming dopaminergic identity. Figure 4D shows the normalized average of importance of features in the FSCV color plot for the estimation of the ratio of serotonin to dopamine. To obtain this, the CNN training was repeated 15 times and after each of the repetitions, each of the samples in the color plot was substituted by a random number between -1 and 1. The ratio change in the predictive error after each of the samples is randomized then used as an estimation of the importance of the sample: the higher the error, the more important the sample is when predicting the ratio of serotonin to dopamine. The CNN chooses which samples from the color plots are important during training to predict the presence of each analyte. The samples of the oxidation peak yield the highest importance in the color plot, although components such as reduction peak also have a non-negligible importance. Figure 4F shows the mean ± SEM predicted ratio of serotonin to dopamine in the CA2 region of the hippocampus of mice for different pharmacological paradigms. First, using an ANCOVA test, we verified that control mice after saline injection (red trace, n = 3 mice) do not show a significant change in the ratio of both analytes over time (*post-hoc* slope F-test, slope = − 2.00e−4 ± 6.92e−8 min^−1^, p = 0.2212). This means that after saline injection, the evoked release of neurotransmitters resembles serotonin release. Following L-DOPA administration (blue trace, n = 5 mice), the CNN model predicts a sustained decrease of the relative ratio of serotonin to dopamine in comparison with control mice after saline injection (*post-hoc t*-test, slope = − 2.00e−3 ± 6.92e−8 min^−1^ *vs.* − 2.00e−4 ± 6.92e−8 min^−1^, p = 0.0001). This effectively means that the evoked release of neurotransmitters is continuously transitioning from serotonin-rich release to a dopamine-rich release in both control and MPTP mice. We believe that this shows that serotoninergic neurons uptake L-DOPA and produce dopamine independent of the number of the dopaminergic cells (at least at these very high concentrations of L-DOPA). For MPTP-treated mice (purple trace, n = 5 mice), the effect of L-DOPA is not significantly different from control mice (*post-hoc t*-test, slope = − 1.80e−3 ± 6.92e−8 min^−1^ vs. − 2.00e−3 ± 6.92e−8 min^−1^, p = 0.8507); a continuous decrease of the relative ratio is present after L-DOPA administration with respect to saline injection in control mice (*post-hoc t*-test, slope = − 1.80e−3 ± 6.92e−8 min^−1^ vs. − 2.00e−4 ± 6.92e−8 min^−1^, p = 0.0004). A mixed ANOVA test between control or MPTP-treated and time after L-DOPA administration was used to underpin the statistical differences between the predicted ratios over time. Importantly, the analysis showed that there was a statistically significant effect of the treatment group in the predicted serotonin to dopamine ratios (mixed ANOVA, F = 4.15, p = 0.0433), as well as in L-DOPA administration (mixed ANOVA, F = 5.67, p < 0.0001).

However, no significance was found for the effect of the interference between both features (one-way ANOVA, F = 0.72, p = 0.7434), suggesting that L-DOPA administration affected both groups of mice similarly. A *Tukey–Kramer post-hoc* paired comparisons test was performed to determine what values were significantly different. For control mice, 80 min is the earliest time after L-DOPA administration when the change of ratio is statistically significant with respect to its control (*post-hoc* paired *t*-test, ratio = 0.97 ± 0.02 *vs.* 0.75 ± 0.01, p = 0.0380). For MPTP-treated mice, this happens at 90 min after L-DOPA administration with respect to the control mice (*post-hoc* paired *t*-test, ratio = 0.97 ± 0.02 *vs.* 0.76 ± 0.07, p = 0.0314). We thus present the real-time conversion of the serotonin signal to dopamine after L-DOPA.

**Fig. 5:**
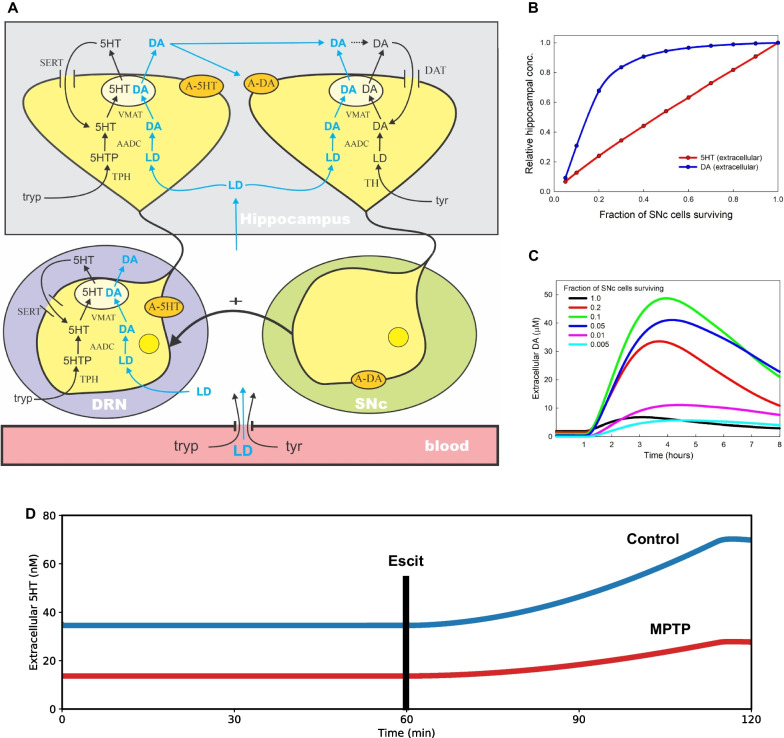
**A** Schematic of SNc dopamine (DA) and DRN serotonin (5HT) innervation of the hippocampus, and the effects of L-DOPA. The model was previously developed in (Reed et al. 2012) and extended here by adding the stimulation of the DRN by the SNc. It is the decrease of this stimulation, as SNc cells die, that causes the decline of serotonin in the hippocampus. **B** Simulation of relative concentrations of serotonin and dopamine in the hippocampus depends on the fractions of surviving SNc cells. **C** The course of extracellular dopamine in the hippocampus after a L-DOPA dose and its dependence on the fraction of SNc cells alive. **D** Simulation of hippocampal extracellular serotonin upon administration of Escit 60 min after the start of the simulation (see “Methods and Materials”). The MPTP trace (red) is computed by reducing the fraction of SNc cells alive to 0.35(Reed et al. 2012)

The final part of this work was to mathematically model the data, based on a previous model (Reed et al. 2012), to show that serotonin is susceptible to MPTP, why SSRIs are not as effective after MPTP and the underlying mechanisms of L-DOPA's effects on serotonin. The model contains the serotonergic projection to the striatum from the DRN as well as the dopaminergic projection to the striatum from the SNc, as well as in internal biochemistry of the synapses (Fig. 5). No detailed structure of the striatum was included in that model, so it can equally well be used for the serotonin projection to the hippocampus from the DRN and the dopamine projection to the hippocampus from the SNc. Only two changes were made to the original model here: (1) the projection from the SNc to the DRN was included (thick black arrow in Fig. 5A), which Guiard showed stimulates DRN firing (Guiard et al. 2008); (2) one parameter was changed so that the ambient normal concentration of serotonin in the hippocampus was 35 nM as measured (Fig. 2) (Reed et al. 2012). The dependence of the DRN firing rate on the SNc cells and the one parameter change are discussed in Methods. Panel B of Fig. 5 shows the concentrations of serotonin and dopamine in the hippocampus computed by the model as the fraction of cells in SNc still alive decreases from 1 to 0.

The dopamine concentration is remarkably homeostatic and does not drop substantially until more than 80% of the SNc cells die. By contrast, the serotonin concentration declines almost linearly as the fraction of SNc cells alive decreases (Panel B in Fig. 5). Our estimate of the number of SNc cells remaining alive (Panel C in Fig. 1) is 30–40%, which means that the model predicts that the serotonin concentration in the hippocampus will be 34–43% of normal, which follows our experimental findings.

An important caveat is the following. We are assuming (see Methods) that the stimulation of all the DRN neurons that project to the hippocampus comes from the SNc. This may be approximately true. According to the classification of DRN neurons in Monti (2010), regions 5 and 6 of the DRN send projections to the hippocampus and the SNc projects to region 5. Thus, all or most of the stimulation of the DRN projection to the hippocampus may come from the SNc. If some stimulation comes from elsewhere, then serotonin in the hippocampus will still descend linearly as SNc cells die, but the slope would not be as steep.

Next, we looked at SSRIs. We added three variables to the model in Reed et al. (2012): E(t), the concentration of Escit in the extracellular space, F(t), the concentration of free SERTs, B(t), the concentration of bound SERTs. Transport across the blood brain barrier has been studied by (Bundgaard et al. 2007) both by experiment and by modelling, and we choose our function, E(t), to have the same shape and half-life as what they found. We choose the amplitude so that at maximum half the SERTs are occupied. The differential equations for the binding of E(t) to the SERTs are described under Methods. Panel D of Fig. 5 shows the results of a model experiment in which a dose of Escit is given at t = 60 min and serotonin is calculated in the extracellular space of the hippocampus both for wild type and MPTP mice. These results are very similar to our experimental results shown in Panel D of Fig. 2.

Finally, we modeled L-DOPA. Panel A of Fig. 5 shows the pathway of L-DOPA's biochemistry in serotonin and dopamine neurons. The details, with experimental references are given in (Reed et al. 2012). This figure shows how L-DOPA can be taken up by serotonin neurons to produce and release dopamine. Panel C of Fig. 5 shows the model dopamine curves in the hippocampus for eight hours after a L-DOPA dose. The black curve is control where the SNc is normal. The dopamine concentration rises from 2 to 6 mM and then declines. If only 20% of the SNc cells remain alive (red) the dopamine pulse is much bigger because there are fewer DATs around to take up the extra dopamine released from the serotonin neurons and there are fewer dopamine autoreceptors to regulate dopamine concentration. This trend continues when only 10% of the SNc neurons remain (green curve), but when the percentage of living SNc cells gets very small (blue, magenta, cyan) the dopamine pulses get smaller. The reason is that as the dopamine terminals become very sparse most of the dopamine diffuses away or is taken up by glial cells or blood vessels. This removal from the system by diffusion is in the model and becomes more and more dominant as the dopamine terminals become sparse.

## Discussion

### Acute MPTP significantly decreases serotonin release

It is well established that experimental PD models are largely driven by dopamine cell loss. This cell loss has directly been investigated via imaging (Boska et al. 2007; Brooks and Pavese 2011; Isaias et al. 2016), and indirectly via chemical methods that showed lower extracellular dopamine (microdialysis) and deficient dopamine signaling (voltammetry) (Garris et al. 1997; Bouchez et al. 2008; Di Giovanni et al. 2009; Heo et al. 2020). Unlike dopamine, serotonergic cell loss is not a unifying phenotype of PD models (Jellinger 1991; Kerenyi et al. 2003; Kish 2003; Politis and Loane 2011). However, there is biochemical and clinical evidence that serotonin plays an important role in PD and in PD models. Post-mortem studies showed that PD patients have lower concentrations of serotonin in the hippocampus both in groups receiving L-DOPA and those that have discontinued use (Scatton et al. 1983). Lewy bodies, the abnormal protein aggregates deposited in neuronal cells during PD, have been shown to accumulate in the serotonergic cell bodies of the raphe nucleus as early as stage two of the disease, suggesting that serotonergic neurons are affected even before dopaminergic neurons (Reijnders et al. 2008). Perhaps the most compelling evidence for the involvement of serotonin in PD is the high comorbidity of the disease with depression, where symptoms often significantly precede motor symptoms (Ishihara and Brayne 2006; Reijnders et al. 2008). Therefore we sought to establish, with our fast voltammetric techniques and mathematical modeling, whether serotonin transmission was impaired in a PD model. We chose the MPTP model as proof of principle. While toxicological models may not provide a true pathogenesis of PD because they cause acute not gradual neurodegeneration, they are a widely accepted model platform for studying the neurochemistry of PD (Tillerson et al. 2002; Greenamyre et al. 2003; Bové et al. 2005; Fornai et al. 2005; Fernagut et al. 2007). Protocols for MPTP administration vary from study to study making it difficult to compare results, however, a common protocol involves acute peripheral injections of a high concentration of MPTP, which was employed here.

We found that animals treated with MPTP had significantly less evoked and ambient serotonin in the CA2 region of the hippocampus than respective controls (Fig. 1). The effect was not significantly different between male and female mice, consistent with prior studies showing no differences in MPTP sensitivity between sexes (Sedelis et al. 2000). Our models show that as SNc cells die, the serotonin concentration drops rapidly and linearly while the dopamine concentration remains quite homeostatic. This is the same idea that we previously presented in the striatum in (Reed et al. 2012) and corresponds to the well-known fact that motor symptoms in PD do not manifest until most (60–90%) of the cells in the SNc have died (Zigmond et al. 1990; Agid 1991; Bergstrom and Garris 2003). In accord, histological analysis of the MPTP-treated tissue showed evidence of dopaminergic cell loss, (Jackson-Lewis et al. 1995; Roostalu et al. 2019) but we found no significant difference in the motor behaviors of MPTP-treated animals in the open field test. This notion and finding is consistent with previous studies reporting no change in locomotion one week following MPTP administration (Willis and Donnan 1987; Nishi et al. 1991; Rousselet et al. 2003). Where more than 20% neurons remained intact.

We previously explained this resilience of the dopamine system in Reed et al. (2009) (see the dopamine curve in Fig. 5B). Suppose, for example, that half the SNc cells have died. Then there is only half as much release in a projection region (the striatum or the hippocampus), but only half as much reuptake via DATs, thus the concentration remains the same. But why does the curve finally come down as the fraction of cells alive becomes small? This is because a released molecule has a lower probability of finding a DAT when the DATs become sparse, and instead diffuses away or is picked up by a blood vessel or a glial cell. This diffusion is part of our model; it plays a very small role when the projection is dense but a much larger role as the projection becomes sparse. In contrast, serotonin is instantly affected by MPTP because the death of SNc cells changes the DRN firing rate (Guiard et al. 2008). The dopaminergic and serotonergic systems are widely accepted to interact and regulate one another. Serotonin receptors have been shown to modulate dopamine activity across multiple brain regions (Alex and Pehek 2007), while dopamine receptors have been shown to indirectly mediate serotonin release in the hippocampus (Saito et al. 1996). As DRN firing goes down, release in the projection region goes down but the number of SERTs remains the same, so the concentration goes down.

In our animals we found no significance in depressive-like and anxiolytic behaviors in MPTP-treated animals using the tail suspension test and elevated zero maze respectively (also supported by previous literature (Vučković et al. 2008)). This is likely due to the insensitivity of behavior to capture serotonergic deficits, for example, we have recently shown that serotonin measurements reliably underpin a chronic stress paradigm without necessarily creating behavioral shifts during tests (Hersey et al. 2022).

In summary, it has been proposed in many clinical and animal studies that depression and/or serotonin dysfunction is an early biomarker for PD (Zygmond 1990; Miquel-Rio et al. 2022) and the novel results here strongly lend support for this idea.

### Escitalopram less able to increase serotonin in MPTP mice

Clinically, PD patients are often prescribed SSRIs to treat highly comorbid depression (Leentjens et al. 2003; Menza et al. 2009). The literature describing the clinical efficacy and safety of SSRIs in PD patients is considerably conflicted. Some clinical studies agree that SSRIs are effective in decreasing non-motor symptoms in PD patients (Ceravolo et al. 2000; Dell’Agnello et al. 2001; Zhuo et al. 2017), while other studies conclude that SSRI efficacy is no different to placebo groups and might worsen PD motor symptoms (Jansen Steur 1993; Simons 1996; Richard et al. 1999; Skapinakis et al. 2010). Reports on adverse effects of SSRIs in PD patients (Zahodne et al. 2012) and a decrease in the therapeutic effect of L-DOPA (Fidalgo et al. 2015) conclude that further studies are required to evaluate the effectiveness of SSRIs in these patients.

We have previously studied the effects of acute administration of Escit in great detail. We found that acute Escit caused a rapid increase in extracellular serotonin levels and decrease in serotonin reuptake rate (Wood and Hashemi 2013; Saylor et al. 2019).

In accord with this prior work, here Escit administration to saline treated animals increased release amplitude and decreased reuptake rate. In our MPTP treated animals, while Escit decreased serotonin reuptake rate, ostensibly there was less effect on serotonin release amplitude. To probe whether this apparent decrease in release amplitude was actually a function of serotonin release, it was necessary to analyze this response with a Michaelis–Menten model. This is because every data point is the result of a balance between the release term [R(t)] and the reuptake term, thus the absolute value is sensitive to both release and reuptake rates. In saline treated animals V_max_ decreased after Escit by 70%; this is expected and consistent with Escit reuptake inhibition (Wood and Hashemi 2013; Wood et al. 2014). The rate of serotonin release after Escit in these animals also decreased (thus the increase in release magnitude is apparent; it does not reflect an increase in serotonin release, but rather the decrease in reuptake). V_max_ is decreased to a lesser extent in MPTP-treated animals (60%), indicating that SERT’s ability to bind Escit may be decreased after MPTP. These results agree with previous studies in both MPTP-induced PD in mice and naturally occurring PD in humans (Kish et al. 2008; Albin et al. 2008; Politis and Loane 2011; Pain et al. 2013). As such, Escit is in general less able to increase extracellular serotonin, evidenced by the FSCAV experiment in Fig. 2, that shows a 32.64 nM increase in basal serotonin after Escit in control animals *vs.* 15.09 nM increase in MPTP-treated animals. We were able to perfectly capture this effect in our mathematical model by simulating MPTP animals via a decrease in viable SNc cells to 35%.

This chemical information may place some of the inconsistency of the clinical data into context.

### L-DOPA induces serotonin-deficits in MPTP mice

We showed that evoked and ambient serotonin is reduced in MPTP-treated animals and that SSRIs are less able to increase serotonin levels. Next, we turned towards L-DOPA treatment since this frontline PD treatment is thought to interact with serotonin (Riahi et al. 2011; Reed et al. 2012; Carta and Tronci 2014; Politis et al. 2014). Specifically, it is thought that at high L-DOPA availability, this precursor is taken up by serotonergic cells, that then produce dopamine, at the expense of serotonin (Tanaka et al. 1999; Nicholson and Brotchie 2002; Maeda et al. 2005; Navailles et al. 2010; Carta and Björklund 2018; Sellnow et al. 2019; Corsi et al. 2021). Using this as a working hypothesis, we expected that after acute L-DOPA, the signal we measure would contain both dopaminergic and serotonergic components and indeed a shift in oxidation potential of the signal is clear in voltammograms post L-DOPA in Fig. 4, implying a shift from serotonin to something else, which we hypothesized to be dopamine. We are not able to quantify the change with conventional calibration techniques since the dopamine and serotonin peaks significantly overlap in the potential domain. Over the past few years, there has been an interest to simultaneously measure serotonin and dopamine with voltammetric techniques by using different electrode materials that separate the peaks (Selvaraju and Ramaraj 2005; Swamy and Venton 2007; Zhou et al. 2013; Castagnola et al. 2021), novel waveforms (Movassaghi et al. 2021) and machine learning methods with in vitro mixture datasets (Bang et al. 2020; Movassaghi et al. 2021). Here we took a novel approach by generating a synthetic, relative dataset from in vivo pharmacologically-validated serotonin and dopamine evoked release signals. We utilized dopamine signals from the striatum, a brain region strongly innervated by dopaminergic neurons (Cachope and Cheer 2014), and serotonin signals from the CA2 region of the hippocampus to create a mixture dataset used to train a CNN to predict the relative ratio of serotonin to dopamine in the signal. After L-DOPA administration, our CNN predicts a sustained decrease of the relative ratio of serotonin to dopamine. Meaning that the evoked release of neurotransmitters is continuously transitioning from serotonin-rich release to dopamine-rich release, showing for the first time, the real-time conversion of the serotonin signal to dopamine after L-DOPA. We modeled L-DOPA’s biochemical pathways in serotonergic cells (Fig. 5). Tyrosine, tryptophan, and L-DOPA are all transported across the blood–brain barrier by the L-type amino acid transporter (Guidotti et al. 1992), and all three are taken up by all cells that express this transporter. Serotonin neurons express tryptophan hydroxylase which adds an OH group to tryptophan, and then aromatic amino acid decarboxylase (AADC) cuts off the carboxyl group to make serotonin. When a serotonin neuron takes up L-DOPA, the AADC cuts off its carboxyl group to make dopamine. Both the serotonin and the dopamine in the cytosol are packaged into the same vesicles by the monoamine transporter. And when the action potential arrives some of these vesicles release their contents (both serotonin and dopamine) into the extracellular space. That is, the serotonin neurons are turned into dopamine-secreting neurons. In further support of our hypothesis, there is independent chemical validation of our findings with microdialysis (Navailles et al. 2013).

This notion (Navailles et al. 2010; Reed et al. 2012; Carta and Tronci 2014) is significant because L-DOPA has been shown to be largely ineffective in treating comorbid depressive symptoms (Cummings 1992; Kim et al. 2009), sometimes significantly worsening these symptoms (Choi et al. 2000; Nègre-Pagès et al. 2010; Hanganu et al. 2014). Moreover the signal after L-DOPA takes longer to clear. This finding could be due to reduced reuptake efficacy, because there are fewer DATs than SERTs in the CA2 region of the hippocampus (Kwon et al. 2008; Dale et al. 2016). Furthermore, serotonin neurons lack the necessary feedback mechanisms to control dopaminergic release from their terminals, resulting in uncontrolled release (Carta and Tronci 2014). This idea has also been put forth in the context of LID, a condition resulting in uncontrolled movements following chronic L-DOPA treatment (Carta and Tronci 2014). Thus, our work may give important new contexts to L-DOPA therapy (with the limitation that we’re working in one brain region). Specifically, that it is important to consider serotonin, even when administering dopamine-specific pharmacology.

In sum, serotonergic neurochemistry has been largely understudied in the context of PD. We evaluated evoked and ambient serotonin experimentally and theoretically in a toxicological model of Parkinsonism and found that mice receiving MPTP had significantly lower evoked and ambient serotonin concentrations than controls. Furthermore, Escit administration was unable to increase serotonin concentrations to levels of control animals. Finally, using a novel neural network we observe, in real-time, an increased ratio of dopamine to serotonin release in the hippocampus following L-DOPA, suggesting that serotonergic neurons release dopamine at the expense of serotonin. These results validate key prior hypotheses about the roles of serotonin during PD and potentially open an avenue of study to improve therapeutics for LID and depression.

## Data Availability

The datasets used and/or analyzed during the current study are available from the corresponding author on reasonable request.

## Abbreviations

AADC: Aromatic l-amino acid decarboxylase
ANCOVA: Analysis of covariance
ANOVA: Analysis of variance
CV: Cyclic voltammogram
CNN: Convolutional neural network
DA: Dopamine
DAT: Dopamine transporter
DRN: Dorsal raphe nucleus
Escit: Escitalopram
FSCAV: Fast-scan controlled-adsorption cyclic voltammetry
FSCV: Fast-scan cyclic voltammetry
L-DOPA: Levodopa
LID: Levodopa induced dyskinesia
MPTP: 1-Methyl-4-phenyl-1,2,3,6-tetrahydropyridine HCl
PD: Parkinson’s disease
RELU: Rectified linear unit
RMSE: Root mean square error
SEM: Standard error of the mean
SELU: Scaled exponential linear unit
SERT: Serotonin transporter
SNc: Substantia nigra pars compacta
SSRI: Selective serotonin reuptake inhibitor
VTA: Ventral tegmental area
5-HT: 5-hydroxytryptamine, serotonin
